# Out-of-hospital cardiac arrest risk attributable to temperature in Japan

**DOI:** 10.1038/srep39538

**Published:** 2017-01-03

**Authors:** Daisuke Onozuka, Akihito Hagihara

**Affiliations:** 1 Department of Health Communication, Kyushu University Graduate School of Medical Sciences, Fukuoka, Japan

## Abstract

Several studies have estimated the associations between extreme temperatures and mortality and morbidity; however, few have investigated the attributable fraction for a wide range of temperatures on the risk of out-of-hospital cardiac arrest (OHCA). We obtained daily records of OHCA cases in the 47 Japanese prefectures between 2005 and 2014. We examined the relationship between OHCA and temperature for each prefecture using a Poisson regression model combined with a distributed lag non-linear model. The estimated prefecture-specific associations were pooled at the nationwide level using a multivariate random-effect meta-analysis. A total of 659,752 cases of OHCA of presumed-cardiac origin met the inclusion criteria. Overall, 23.93% (95% empirical confidence interval [eCI]: 20.15–26.19) of OHCA was attributable to temperature. The attributable fraction to low temperatures was 23.64% (95% eCI: 19.76–25.87), whereas that of high temperatures was 0.29% (95% eCI: 0.21–0.35). The attributable fraction for OHCA was related to moderate low temperature with an overall estimate of 21.86% (95% eCI: 18.10–24.21). Extreme temperatures were responsible for a small fraction. The majority of temperature-related OHCAs were attributable to lower temperatures. The attributable risk of extremely low and high temperatures was markedly lower than that of moderate temperatures.

Temperature-related mortality and morbidity is a growing public health concern^1^. High and low temperatures are associated with an increased risk of mortality and morbidity in a wide range of climates and countries, and extreme temperature-related mortality is expected to increase as the frequency, intensity, and duration of extreme events increase due to climate change^2^,^3^. Most previous studies focused on extreme weather events or aimed to identify exposure-response associations between temperature and mortality. For example, an association between extremely low and high temperatures and a significant increase in all-cause, cardiovascular and respiratory diseases has been reported^4^. However, few quantitative studies have investigated the risk of out-of-hospital cardiac arrest (OHCA) related to temperature.

OHCA is an on-going public health issue with a high case fatality rate and associated with both patient and environmental factors. Previous studies have shown that patient characteristics such as gender, age, weight, diet, smoking, physical activity, socioeconomic status, family history of cardiac arrest, medical history of heart disease, race, and emergency medical services (EMS) are associated with incidence/survival of OHCA^5^,^6^,^7^,^8^. Additionally, emerging evidence suggests that both high and low temperatures may be an important risk factor for OHCA^9^,^10^,^11^,^12^. However, most previous studies evaluated the association between OHCA and temperature in terms of relative risk, and few studies have investigated the potential disease burden of OHCA using attributable risk, such as absolute excess (numbers) or relative excess (fraction) of OHCA. The attributable fraction and the absolute number can quantify the preventable public health burden due to a specific risk factor^13^,^14^,^15^,^16^,^17^. Thus, estimating the OHCA burden attributable to temperature is essential for the development of strategies to prevent or control temperature-related OHCA.

We investigated the population attributable risk of OHCA due to temperature and the relative contributions of low and high temperatures over a 10-year period in the 47 Japanese prefectures using flexible and advanced statistical approaches based on multivariate meta-regression models with time-varying distributed lag non-linear models^14^,^15^,^16^,^17^,^18^,^19^,^20^,^21^,^22^,^23^. To the best of our knowledge, our study is the first to evaluate the attributable risk of OHCA due to temperature using national data from a comprehensive sample of OHCA cases in Japan.

## Results

### Descriptive analysis

**Figure 1:**
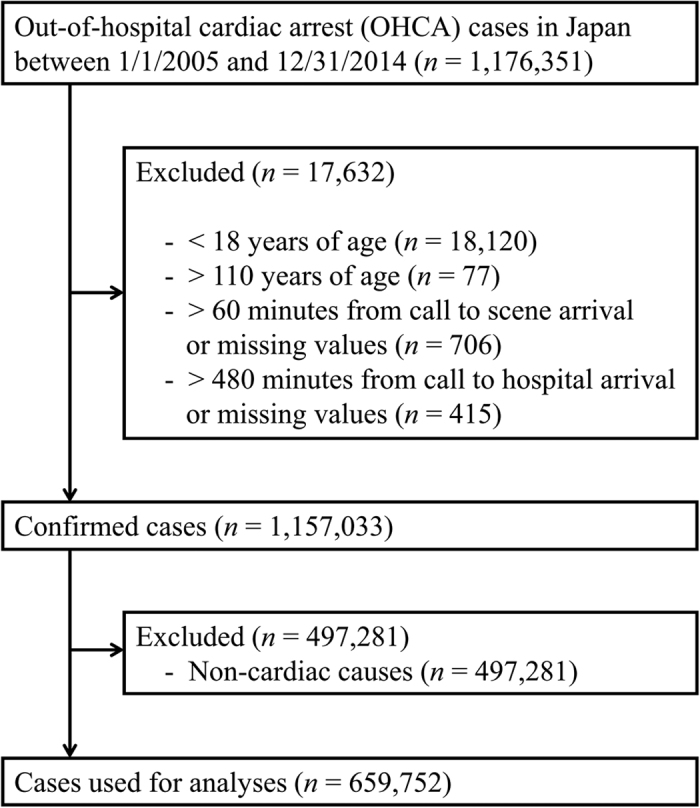
Out-of-hospital cardiac arrest (OHCA) data included in the study.

A total of 1,176,351 cases of OHCA occurred between January 1, 2005 and December 31, 2014 in the 47 Japanese prefectures. Of those, we evaluated 659,752 cases of OHCA of presumed-cardiac origin that met the inclusion criteria (Fig. 1, Table 1). The daily mean temperature was 15.5 °C, and we found a broad range of temperatures among the various prefectures. The prefecture-specific daily mean temperature ranged from 9.4 °C in Hokkaido Prefecture to 23.2 °C in Okinawa Prefecture (Table 1).

### Exposure-response associations

**Figure 2:**
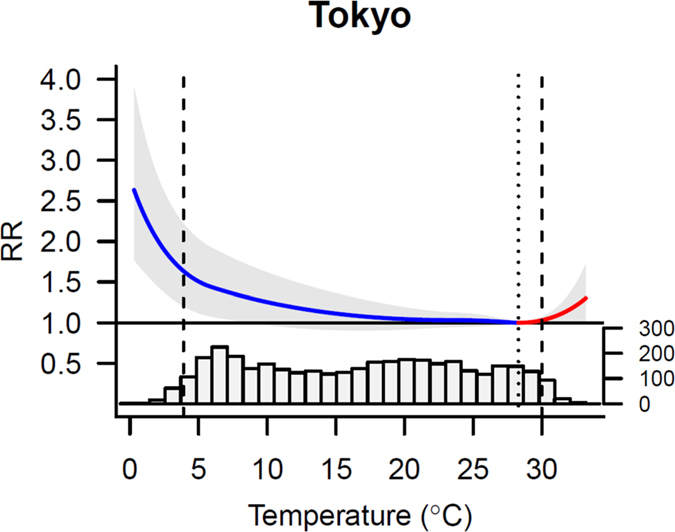
Overall cumulative exposure-response associations between the relative risks (95% CI) for OHCA and temperatures in Tokyo Prefecture. Exposure-response associations expressed as the best linear unbiased prediction (with a 95% emperical confidence interval, shaded grey) with the related temperature distributions. Vertical lines represent the percentile of minimum morbidity temperature (dotted) and the 2.5th and 97.5th percentiles of the temperature distribution (dashed).

Tokyo Prefecture had the largest population and, thus, was selected as a representative prefecture. Figure 2 shows the overall cumulative exposure-response curves (best linear unbiased predictions) for the relative risk of OHCA and temperatures in Tokyo Prefecture with the corresponding minimum morbidity temperature (MMT) and cutoffs used to define extreme temperatures. The morbidity risks for OHCA increased slowly and linearly for temperatures below the MMT. Corresponding graphs for all 47 prefectures are included in Supplementary Figure S1.

### Attributable risk

The attributable risks related to low and high temperatures according to prefecture are shown in Table 2. The median MMT was at the 92nd percentile at the national level. The prefecture-specific MMT ranged from the 76th percentile in Okinawa to the 99th percentile in Kagoshima, consistent with the diverse range of climatic conditions by prefecture. Overall, 23.93% (95% eCI: 20.15–26.19) of OHCAs were attributable to non-optimal temperature. Low temperature was responsible for most of the OHCA burden; the attributable risk of OHCA for low temperatures was 23.64% (95% eCI: 19.76–25.87). In contrast, the fraction attributable to high temperature was 0.29% (95% eCI: 0.21–0.35). The multivariate Cochran’s Q test revealed that geographical heterogeneity between prefectures was not significant (*p* = 0.324; *I*^*2*^ = 3.9%).

The pooled attributable risks due to temperatures stratified by gender and age are shown in Table 3. In the gender-stratified analysis, the attributable risk of OHCA for male was 21.12% (95% eCI: 16.54–23.88); whereas an identical estimate for female was 26.86% (95% eCI: 22.54–29.08). In the age-stratified analysis, the attributable risks due to temperature were 17.93% (95% eCI: 8.82–20.89) in the 18–64-year age group, 25.24% (95% eCI: 16.39–28.88) in the 65–74-year age group, and 28.39% (95% eCI: 25.87–30.12) in the 75–110-year age group.

**Figure 3:**
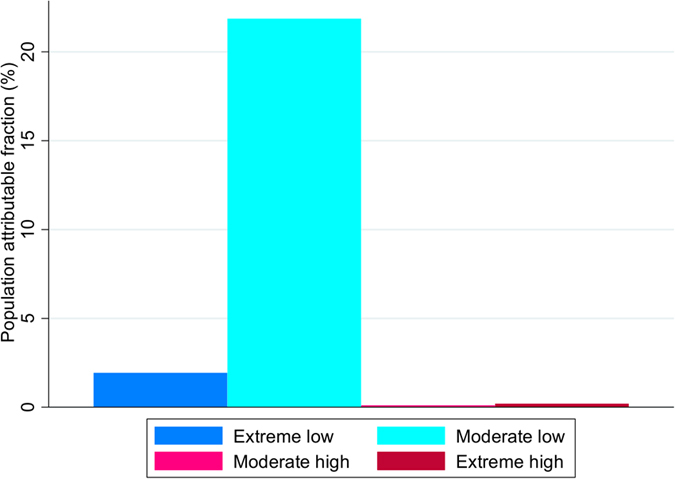
Cumulative population attributable fraction of OHCA due to moderately and extremely low and high temperature. Extremely low temperatures were defined as temperatures below the 2.5th prefecture-specific percentile of temperature, and extremely high temperatures were defined as temperatures above the 97.5th prefecture-specific percentile of temperature. Moderately low temperatures were defined as the ranges between the 2.5th prefecture-specific percentile of temperature and the minimum morbidity temperature (MMT), and moderately high temperatures were defined as the ranges between the MMT and 97.5th prefecture-specific percentile of temperature.

The cumulative population attributable fraction related to moderate and extreme temperatures are shown in Fig. 3, and attributable fractions according to prefecture are shown in Supplementary Table S1. The attributable fraction of moderately low temperature for the risk of OHCA had an overall risk of 21.86% (95% eCI: 18.10–24.21). Extreme temperatures were responsible for a small fraction of risk: 1.92% (95% eCI: 1.73–2.01) for extremely low temperature and 0.20% (95% eCI: 0.15–0.23) for extremely high temperature.

The sensitivity analysis revealed that varying the choice of model had little effect on the estimates (Supplementary Table S2).

## Discussion

Our nationwide study produced several notable findings. Most importantly, our results showed that temperature accounted for a substantial fraction of OHCAs, and that most of morbidity burden was attributable to low temperatures. Specifically, most of the attributable OHCAs were caused by moderately low temperatures, whereas the contribution of extremely high and low temperatures was relatively small.

We found that most of the OHCA risk attributable to temperature was related to low temperatures. Despite increased risk of OHCA due to extreme temperatures, most of OHCA burden was attributable to moderately low temperatures. Our findings are consistent with those of previous studies showing that sudden cardiac death increased in winter^9^. A previous study found that mortality attributable to heat was between 0.37% and 1.45% in London, Budapest, and Milan^24^. Furthermore, a study conducted in London found that the attributable fraction per 1 °C drop below 15 °C was 5.4% between 1986 and 1996, whereas no deaths attributable to heat were recorded during that period^25^. Furthermore, a recent multinational observational study found that cold-related deaths outnumbered heat-related deaths, and also that mortality attributable to moderate non-optimal temperatures was substantially greater than that due to extreme temperatures^14^. Our findings suggest that non-optimal temperatures are associated with a substantial increase in OHCAs.

An association between cardiovascular events and temperature is supported by previous studies showing that hospitalization for cardiovascular disease increases when temperatures are low^26^, and that high ambient temperature is negatively associated with emergency ambulance transport for aneurysm, hemorrhagic stroke, and hypertension^27^. A recent study found that circulatory and coronary heart disease and ST-elevation myocardial infarction (STEMI) mortality increased as temperatures decreased^28^. It may be that low temperatures precipitate sympathetic stimulation and increase cardiac workload, which in turn may stress a patient with severe coronary stenosis and/or advanced heart failure beyond the point of compensation^28^,^29^,^30^. Low temperatures may contribute to the cardiovascular stress response by increasing blood viscosity, changing heart rate variability, and affecting inflammatory responses^31^. High excess risks of heart failure, arrhythmia, and atrial fibrillation have been reported during low temperature periods^32^. Cold temperatures increase sympathetic tone, blood pressure, vascular resistance, fibrinogen level, platelet count, certain clotting factors, and blood viscosity, which in turn increases the risk of plaque rupture, thrombosis, and STEMI mortality^28^,^29^,^30^. Furthermore, individuals with lower vitamin D levels are susceptible to sudden cardiac death during mild winter temperatures; thus, vitamin D deficiency resulting from reduced sun exposure during winter may be a key contributor to sudden cardiac death^33^,^34^,^35^. The precise mechanism underlying the association between temperature and OHCA is unclear, and our findings highlight the need for further investigation of this relationship.

Low temperatures trigger bronchoconstriction and impair the mucociliary clearance system, and other immune responses, resulting in local and systemic inflammation and increased risk for respiratory infections^36^. The physiological mechanisms activated by low temperatures may persist longer than those triggered by high temperatures^31^, and most of the attributable risks of OHCA occur on moderately cold days. Furthermore, the compensatory effects suggest that cold-related mortality may have the greatest impact on individuals whose health is already compromised and often terminal^37^.

A random-effect multivariate meta-analysis revealed no significant geographical heterogeneity in the temperature-OHCA relationships between prefectures. Our results are consistent with a previous meta-analysis, which indicated that latitude had little effect on cardiovascular morbidity^38^. Our results may support the existence of population acclimatization, suggesting that populations adapt to local weather patterns in terms of physiology, behavior, and technology. Furthermore, we evaluated regional heterogeneity by the multivariate extension of the Cochran Q test and *I*^*2*^ index. However, it is worth noting that both are dependent on the power of the analysis, with the *I*^*2*^ index increasing and the p-value of the test decreasing proportionally to the precision of the estimates^39^. Because of low numbers of OHCA events in some prefectures, the power to detect geographical heterogeneity of temperature effects might be limited. Thus, we should bear in mind when evaluating heterogeneity.

Our findings suggest that a better understanding of the relationships between high and low temperatures and OHCA is essential for the identification of specific factors that affect susceptibility to high and low temperatures. In Japan, the demand for emergency ambulances has nearly exceeded capacity, forcing public health officials to reduce the use of ambulances and develop mitigation strategies^40^. Our findings may help public health officials predict temperature-related OHCA to prepare for the effects of climatic change through public health intervention strategies, such as timely public health and medical advice, and improvements to housing and urban planning, early warning systems, health education, and healthcare system preparedness^41^.

Our study had several limitations. First, we used an observational design. However, because the registration of OHCA cases was reasonably comprehensive, we are confident that there were no major selection biases. Nevertheless, we acknowledge that observational studies only partially control and adjust for the factors actually measured, and as such we could not control for unknown confounding factors or prevent bias. Second, we did not control for air pollution. A recent study found that PM_2.5_ concentration was associated with ischemic heart disease mortality and morbidity^42^. However, the results of a recent multicountry study suggested that not controlling for air pollution was unlikely to have materially altered the pattern of results^14^. Moreover, a previous study found an association between elevated apparent temperatures and increased mortality with no confounding or effect modification attributable to air pollution^43^. Further investigation of the effects of air pollution and temperature on OHCA is warranted. Third, we did not consider factors such as acclimatization, susceptibility, resilience, socioeconomic status, community characteristics, or the proportion of the population consisting of unwell individuals. Additionally, weather conditions such as temperature have an influence on road conditions which may be issue among OHCAs since patient treatment in the pre-hospital setting and transport to hospital are key. These factors could influence the effects of temperatures on OHCA and indicate the need for more precise modeling in future studies. Fourth, we used all presumed cardiac etiology cases in this study. However, a recent study has suggested that there is wide variation in how presumed cardiac etiology is codified between communities^44^. The CARES registry in the US began using all-non traumatic etiology moving away from presumed cardiac etiology since 2013^44^. This more inclusive classification is not considered as the standard for the Utstein template, and our findings highlight the need for using all non-traumatic etiology cases rather than the more subject presumed cardiac etiology.

In conclusion, despite its limitations, our study showed that most of the temperature-related OHCA burden was attributable to low temperatures. The effect of extreme temperatures was substantially less than that of moderate temperatures. Our findings suggest that public health interventions to control temperature-related OHCA should take a wide range of temperatures into account.

## Methods

### Study design and data

Detailed information on EMS system in Japan is published elsewhere^45^,^46^. Briefly, municipal governments provide emergency medical services through approximately 800 fire stations with dispatch centers under the Fire Service Act. As EMS providers are not allowed to terminate resuscitation in the field, all patients with OHCA who are treated by EMS personnel are then transported to hospitals^47^,^48^. Following the standardized Utstein-style reporting guidelines for cardiac arrest, the EMS personnel summarize each case of OHCA in cooperation with the physicians in charge^47^,^48^. OHCA data for the 47 Japanese prefectures between 2005 and 2014 were obtained from the Japanese Fire and Disaster Management Agency of the Ministry of Internal Affairs and Communications. In Japan, the registration of OHCA episodes is required under the Fire Service Act, which is considered to be comprehensive. Cases were assessed for enrollment in our study according to the inclusion criteria (Fig. 1).

Daily mean temperatures and relative humidity data collected by representative monitoring stations in each prefecture were obtained from the Japan Meteorological Agency. Daily mean temperature was selected as the main exposure index because it reflects exposure throughout the day than maximum or minimum temperatures, which occur instantaneously, and can be readily interpreted for decision-making purposes^49^,^50^.

### Ethical Approval

Our study was approved by the Institutional Ethics Committee of Kyushu University Graduate School of Medical Sciences. This was an observational study using national registry data; thus, the requirement for written informed consent was waived.

### Statistical analysis

We conducted a two-stage time-series analysis using data from 47 Japanese prefectures to investigate the OHCA risk attributable to temperature. In the first stage, we estimated prefecture-specific temperature–OHCA relationships using a time-series regression model, which allowed for nonlinearity and delayed effects. In the second stage, a multivariate meta-regression analysis was used to pool the prefecture-specific estimates at the national level.

### First-stage time series model

We first examined the association between temperature and OHCA in each prefecture using a time-series quasi-Poisson regression model combined with a distributed lag non-linear model^18^,^19^,^21^. The distributed lag non-linear model was used to control for the non-linear and delayed effect of daily mean temperature. A natural cubic B-spline of time with a smoothing function of 8 degrees of freedom (df) per year was used to control for seasonality and long-term trends. The exposure-response curve was modelled with a quadratic B-spline with three internal knots placed at the 10th, 75th, and 90th percentiles of the prefecture-specific temperature distributions. The lag-response curve was also modelled with a natural cubic B-spline with an intercept and three internal knots placed at equally spaced values in the log scale. Because there is no universally accepted setting for distributed lag non-linear models, these choices of the degree of freedom and knots were motivated by previous studies^14^. A categorical variable for day of the week, and an indicator variable for public holidays, were also included in the model. Lag periods up to 21 days were assessed to capture the delayed effects of low temperatures and excluded cases that were a few days ahead. The choice of 21 days for lag periods was motivated by previous studies^14^.

Associations between prefecture and temperature-induced morbidity are typically evaluated using an absolute temperature scale; however, because the range and distribution of temperatures varied across the 47 prefectures, combining temperature-OHCA curves across prefectures using non-overlapping temperature ranges was problematic. Moreover, relative temperature scales may provide a more reliable indicator of the overall effect of temperature on OHCA than absolute scales because they reflect adaptation to climate change over time and space. Thus, the associations between temperature and OHCA were evaluated using a relative temperature scale according to percentiles of the prefecture-specific mean temperature distribution.

### Second-stage meta-analysis

The estimated prefecture-specific associations were then pooled using multivariate meta-regression models obtained following reduction of the time-series regression model to estimate the non-linear temperature–OHCA relationship at the national level^22^. Multivariate random-effects meta-regression analyses were performed to assess national pooled estimates based on maximum likelihood. Residual inter-prefecture heterogeneity was evaluated using a multivariate extension of Cochran’s Q test and the *I*^*2*^ index^20^.

Fitted multivariate random-effects meta-regression models were used to estimate the best linear unbiased prediction of the overall cumulative associations between temperature and OHCA in each prefecture. The optimal linear unbiased prediction represented a trade-off between the prefecture-specific association tested by the time-series regression model and the prefecture-pooled association. This approach enabled areas with a small number of daily cases or a short time-series to use information from larger populations sharing similar characteristics^14^,^20^.

The temperature with the lowest risk of OHCA morbidity was defined as the MMT. The MMT, which corresponds to a minimum morbidity percentile (MMP) between the 1st and 99th percentiles, was derived from the best linear unbiased prediction of the overall cumulative association between temperature and OHCA in each prefecture. We used the MMT as the reference for calculating the attributable risk by re-centering the quadratic B-spline. We referred to this value as the optimal temperature. This method has been described in detail elsewhere^14^,^23^.

The total attributable number of OHCAs due to non-optimal temperature was calculated by the sum of the contributions from all days of the series. The ratio with the total number of OHCAs produces the total attributable fraction. The components attributable to low and high temperatures were calculated by accumulating the subsets corresponding to days with temperatures lower or higher than the MMT. These components were further divided into moderate and extreme components. Extremely low temperatures were defined as temperatures below the 2.5th prefecture-specific percentile of temperature, and extremely high temperatures were defined as temperatures above the 97.5th prefecture-specific percentile of temperature^14^. These cut-off values were chosen according to previous definitions of extreme temperatures^24^,^50^. Moderately low temperatures were defined as the ranges between the 2.5th prefecture-specific percentile of temperature and the MMT, and moderately high temperatures were defined as the ranges between the MMT and 97.5th prefecture-specific percentile of temperature. Empirical confidence intervals (eCIs) were calculated using Monte Carlo simulations based on a multivariate normal distribution of the best linear unbiased predictions of the reduced coefficients. We used 999 Monte Carlo replications to estimate the significance levels of the results. This method is described in detail elsewhere^14^,^23^.

For the sensitivity analysis, modeling selections were tested by controlling for different df for seasonal and long-term trends (6 and 10 df per year) and relative humidity in the 47 prefectures. All statistical analyses were performed with R software (ver. 3.2.5) using the *dlnm* and *mvmeta* packages (R Core Team, R Foundation for Statistical Computing, Vienna, Austria).

## Additional Information

**How to cite this article**: Onozuka, D. and Hagihara, A. Out-of-hospital cardiac arrest risk attributable to temperature in Japan. *Sci. Rep.* **7**, 39538; doi: 10.1038/srep39538 (2017).

**Publisher's note:** Springer Nature remains neutral with regard to jurisdictional claims in published maps and institutional affiliations.

## Supplementary Material

Supplementary Information

## Figures and Tables

**Table 1:** Population, total number of OHCAs of presumed-cardiac origin, characteristics of OHCAs by gender and age, and mean daily temperature distribution (°C) of the 47 Japanese prefectures between 2005 and 2014.

Prefecture	Population (2010)	Total OHCAs			Age, no. (%)						Mean daily temperature (°C)						
			Male, no. (%)		18–64 years		65–74 years		75–110 years		Mean	SD	Min	2.5th	Median	97.5th	Max
Hokkaido	5,506,419	27,573	11,364	(41.2)	5,808	(21.1)	5,518	(20.0)	16,247	(58.9)	9.4	9.7	−10.7	−6.3	9.6	25.1	29.3
Aomori	1,373,339	9,315	3,958	(42.5)	1,763	(18.9)	1,633	(17.5)	5,919	(63.5)	10.7	9.2	−7.4	−4.2	11.1	26.3	30.1
Iwate	1,330,147	9,411	4,099	(43.6)	1,473	(15.7)	1,570	(16.7)	6,368	(67.7)	10.6	9.6	−7.7	−4.3	10.8	26.5	29.3
Miyagi	2,348,165	13,524	5,852	(43.3)	2,432	(18.0)	2,272	(16.8)	8,820	(65.2)	12.8	8.5	−4.5	−0.7	13.2	27.3	31.2
Akita	1,085,997	7,762	3,542	(45.6)	1,140	(14.7)	1,115	(14.4)	5,507	(70.9)	12.1	9.3	−5.5	−2.3	12.1	27.3	31.6
Yamagata	1,168,924	8,043	3,556	(44.2)	1,223	(15.2)	1,235	(15.4)	5,585	(69.4)	12.0	9.5	−5.8	−2.7	12.1	27.8	30.4
Fukushima	2,029,064	14,778	6,449	(43.6)	2,492	(16.9)	2,361	(16.0)	9,925	(67.2)	13.4	9.0	−4.2	−0.6	13.7	28.8	31.4
Ibaraki	2,969,770	16,466	6,877	(41.8)	3,173	(19.3)	2,996	(18.2)	10,297	(62.5)	14.1	8.3	−1.7	1.2	14.4	28.1	31.0
Tochigi	2,007,683	12,507	5,428	(43.4)	2,406	(19.2)	2,347	(18.8)	7,754	(62.0)	14.4	8.6	−2.5	0.7	14.8	28.6	31.7
Gunma	2,008,068	11,289	4,907	(43.5)	2,085	(18.5)	2,148	(19.0)	7,056	(62.5)	15.0	8.7	−1.4	1.4	15.3	29.6	32.6
Saitama	7,194,556	36,208	15,299	(42.3)	7,400	(20.4)	7,738	(21.4)	21,070	(58.2)	15.5	8.6	−0.8	2.1	15.9	29.9	33.7
Chiba	6,216,289	29,475	12,336	(41.9)	6,004	(20.4)	6,002	(20.4)	17,469	(59.3)	16.3	7.8	0.3	3.8	16.8	29.2	32.1
Tokyo	13,159,388	71,231	29,472	(41.4)	15,262	(21.4)	13,695	(19.2)	42,274	(59.3)	16.7	8.0	0.3	3.9	17.2	30.0	33.2
Kanagawa	9,048,331	46,063	19,568	(42.5)	9,100	(19.8)	8,810	(19.1)	28,153	(61.1)	16.3	7.7	0.3	3.9	16.7	29.0	32.2
Niigata	2,374,450	13,151	5,571	(42.4)	2,109	(16.0)	2,107	(16.0)	8,935	(67.9)	14.1	8.9	−2.8	0.4	14.1	29.0	31.5
Toyama	1,093,247	4,919	1,986	(40.4)	866	(17.6)	878	(17.8)	3,175	(64.5)	14.5	9.0	−2.8	0.2	14.9	29.4	31.7
Ishikawa	1,169,788	4,682	1,860	(39.7)	905	(19.3)	848	(18.1)	2,929	(62.6)	14.9	8.8	−1.8	0.9	15.3	29.5	31.7
Fukui	806,314	3,302	1,329	(40.2)	586	(17.7)	581	(17.6)	2,135	(64.7)	14.8	9.0	−1.8	0.7	15.2	29.7	31.9
Yamanashi	863,075	5,541	2,427	(43.8)	960	(17.3)	926	(16.7)	3,655	(66.0)	15.1	8.8	−2.1	1.1	15.6	29.0	31.8
Nagano	2,152,449	11,267	4,667	(41.4)	1,721	(15.3)	1,855	(16.5)	7,691	(68.3)	12.2	9.6	−6.0	−2.9	12.6	27.3	30.0
Gifu	2,080,773	13,887	5,959	(42.9)	2,212	(15.9)	2,373	(17.1)	9,302	(67.0)	16.2	8.8	−1.7	2.0	16.6	30.3	32.7
Shizuoka	3,765,007	19,348	8,124	(42.0)	3,432	(17.7)	3,570	(18.5)	12,346	(63.8)	16.9	7.5	1.7	4.6	17.3	28.9	31.9
Aichi	7,410,719	38,712	16,615	(42.9)	7,106	(18.4)	7,400	(19.1)	24,206	(62.5)	16.2	8.7	−1.5	2.3	16.6	30.1	32.7
Mie	1,854,724	11,473	4,790	(41.8)	1,866	(16.3)	2,047	(17.8)	7,560	(65.9)	16.3	8.3	−0.3	3.2	16.5	29.8	33.1
Shiga	1,410,777	6,279	2,727	(43.4)	1,075	(17.1)	1,010	(16.1)	4,194	(66.8)	15.0	8.7	−1.6	1.5	15.1	29.3	31.8
Kyoto	2,636,092	13,885	6,166	(44.4)	2,322	(16.7)	2,558	(18.4)	9,005	(64.9)	16.1	8.8	−1.2	2.3	16.4	30.3	32.0
Osaka	8,865,245	45,420	19,413	(42.7)	9,563	(21.1)	9,801	(21.6)	26,056	(57.4)	17.1	8.4	0.5	3.7	17.4	30.5	32.7
Hyogo	5,588,133	25,495	11,039	(43.3)	4,754	(18.6)	4,847	(19.0)	15,894	(62.3)	17.0	8.3	−0.3	3.6	17.6	29.9	32.5
Nara	1,400,728	7,778	3,456	(44.4)	1,290	(16.6)	1,530	(19.7)	4,958	(63.7)	15.1	8.6	−1.1	1.7	15.3	28.8	30.5
Wakayama	1,002,198	5,290	2,334	(44.1)	874	(16.5)	1,047	(19.8)	3,369	(63.7)	16.9	8.2	0.9	3.8	17.3	29.5	32.7
Tottori	588,667	3,983	1,719	(43.2)	692	(17.4)	654	(16.4)	2,637	(66.2)	15.2	8.7	−2.0	1.2	15.4	29.6	32.0
Shimane	717,397	4,955	2,292	(46.3)	739	(14.9)	773	(15.6)	3,443	(69.5)	15.2	8.4	−1.6	1.7	15.4	29.2	32.2
Okayama	1,945,276	9,003	3,896	(43.3)	1,680	(18.7)	1,540	(17.1)	5,783	(64.2)	16.5	8.7	−0.9	2.8	16.8	30.4	32.3
Hiroshima	2,860,750	11,001	4,557	(41.4)	2,125	(19.3)	2,023	(18.4)	6,853	(62.3)	16.4	8.6	−2.0	2.8	16.9	30.1	31.8
Yamaguchi	1,451,338	7,295	3,182	(43.6)	1,221	(16.7)	1,357	(18.6)	4,717	(64.7)	15.8	8.6	−2.1	1.9	16.2	29.3	31.0
Tokushima	785,491	3,192	1,232	(38.6)	709	(22.2)	647	(20.3)	1,836	(57.5)	16.8	8.1	−0.4	3.8	17.3	29.5	32.6
Kagawa	995,842	4,793	2,054	(42.9)	890	(18.6)	779	(16.3)	3,124	(65.2)	16.8	8.5	−0.1	3.5	17.1	30.4	33.0
Ehime	1,431,493	9,035	3,962	(43.9)	1,517	(16.8)	1,502	(16.6)	6,016	(66.6)	16.8	8.2	−0.4	3.7	17.2	29.7	31.7
Kochi	764,456	3,604	1,567	(43.5)	640	(17.8)	620	(17.2)	2,344	(65.0)	17.4	7.8	−0.1	4.1	18.0	29.4	31.3
Fukuoka	5,071,968	15,892	6,818	(42.9)	3,392	(21.3)	2,998	(18.9)	9,502	(59.8)	17.4	8.0	−0.1	4.1	17.9	30.5	32.8
Saga	849,788	3,391	1,431	(42.2)	620	(18.3)	583	(17.2)	2,188	(64.5)	16.9	8.4	−0.8	3.0	17.4	30.0	32.3
Nagasaki	1,426,779	6,142	2,603	(42.4)	1,181	(19.2)	1,114	(18.1)	3,847	(62.6)	17.4	7.8	−0.3	4.1	17.9	29.5	31.9
Kumamoto	1,817,426	8,968	3,935	(43.9)	1,543	(17.2)	1,489	(16.6)	5,936	(66.2)	17.3	8.4	−0.7	3.1	17.9	30.0	31.7
Oita	1,196,529	5,222	2,073	(39.7)	1,009	(19.3)	960	(18.4)	3,253	(62.3)	16.8	7.9	−0.3	4.0	17.2	29.5	31.7
Miyazaki	1,135,233	4,965	2,204	(44.4)	920	(18.5)	889	(17.9)	3,156	(63.6)	17.6	7.5	0.8	4.8	18.2	29.3	31.6
Kagoshima	1,706,242	8,487	3,795	(44.7)	1,546	(18.2)	1,456	(17.2)	5,485	(64.6)	18.8	7.6	0.7	5.6	19.4	30.1	31.7
Okinawa	1,392,818	5,750	2,449	(42.6)	1,648	(28.7)	1,017	(17.7)	3,085	(53.7)	23.2	4.8	10.3	14.4	23.5	29.8	31.1

**Table 2:** Attributable fraction for OHCA according to prefecture expressed as total and separate components of low and high temperatures.

Prefecture	MMP	MMT (°C)	Total (%)		Low temperature (%)		High temperature (%)	
Hokkaido	94th	23.4	27.28	(3.74, 42.15)	26.75	(3.25, 43.26)	0.53	(0.16, 0.81)
Aomori	92nd	23.6	36.33	(18.27, 48.52)	35.62	(17.22, 48.34)	0.70	(0.22, 1.12)
Iwate	92nd	24.0	30.53	(7.16, 45.79)	29.71	(8.84, 46.10)	0.82	(0.34, 1.24)
Miyagi	93rd	25.2	22.26	(1.26, 36.96)	21.78	(−0.05, 37.00)	0.48	(0.20, 0.72)
Akita	93rd	25.3	28.66	(7.37, 43.09)	28.29	(9.03, 42.55)	0.37	(0.13, 0.61)
Yamagata	92nd	25.3	31.21	(11.55, 44.90)	30.50	(11.42, 44.59)	0.71	(0.28, 1.09)
Fukushima	92nd	26.2	33.02	(17.80, 45.39)	32.54	(17.32, 44.06)	0.48	(0.09, 0.79)
Ibaraki	89th	25.1	25.30	(3.42, 40.45)	24.77	(2.96, 40.15)	0.54	(−0.02, 1.00)
Tochigi	92nd	26.6	21.27	(1.84, 36.50)	20.89	(1.46, 34.41)	0.38	(0.12, 0.60)
Gunma	92nd	27.4	27.93	(12.11, 39.94)	27.61	(11.63, 40.56)	0.31	(0.04, 0.58)
Saitama	92nd	27.8	23.95	(8.64, 35.84)	23.71	(6.90, 35.34)	0.23	(−0.01, 0.45)
Chiba	92nd	27.7	19.39	(2.31, 32.09)	19.25	(0.50, 33.04)	0.15	(−0.10, 0.37)
Tokyo	92nd	28.3	15.02	(0.97, 26.84)	14.85	(−1.25, 26.72)	0.17	(−0.03, 0.37)
Kanagawa	90th	27.0	18.27	(2.77, 31.54)	18.08	(2.34, 31.23)	0.19	(−0.10, 0.46)
Niigata	92nd	26.5	30.79	(13.42, 42.25)	30.33	(11.54, 42.13)	0.46	(0.07, 0.79)
Toyama	92nd	27.2	30.16	(14.02, 42.16)	29.72	(12.82, 41.89)	0.45	(0.09, 0.77)
Ishikawa	91st	27.2	29.05	(10.41, 42.40)	28.67	(8.60, 41.84)	0.38	(0.03, 0.69)
Fukui	91st	27.3	30.09	(12.06, 42.66)	29.66	(10.93, 42.49)	0.43	(0.05, 0.77)
Yamanashi	92nd	27.4	26.07	(6.34, 40.53)	25.82	(6.95, 39.87)	0.25	(0.03, 0.46)
Nagano	91st	25.3	37.46	(22.01, 48.42)	36.94	(20.50, 48.22)	0.52	(0.14, 0.86)
Gifu	93rd	28.7	21.78	(−0.32, 36.69)	21.54	(−0.24, 35.90)	0.24	(−0.12, 0.56)
Shizuoka	82nd	25.2	21.05	(−1.71, 36.58)	20.68	(−0.89, 35.24)	0.37	(−0.74, 1.27)
Aichi	93rd	28.7	27.46	(11.29, 39.72)	27.31	(11.38, 39.82)	0.15	(−0.19, 0.43)
Mie	92nd	28.1	28.56	(9.63, 41.43)	28.43	(10.12, 40.98)	0.13	(−0.15, 0.37)
Shiga	89th	26.9	28.46	(10.04, 41.44)	28.10	(7.38, 41.20)	0.36	(0.02, 0.67)
Kyoto	91st	28.5	30.22	(13.92, 41.60)	29.99	(14.60, 41.72)	0.23	(−0.14, 0.52)
Osaka	92nd	29.1	13.52	(−1.97, 27.35)	13.32	(−4.35, 26.37)	0.20	(−0.06, 0.46)
Hyogo	99th	30.4	32.89	(15.01, 46.11)	32.89	(15.95, 45.68)	0.00	(−0.05, 0.04)
Nara	83rd	24.9	31.99	(11.16, 46.45)	31.18	(11.05, 44.97)	0.81	(−0.29, 1.75)
Wakayama	90th	28.0	21.84	(−0.29, 37.60)	21.74	(0.39, 36.38)	0.10	(−0.17, 0.34)
Tottori	92nd	27.7	23.30	(1.08, 39.06)	22.95	(2.93, 38.41)	0.36	(0.08, 0.60)
Shimane	92nd	27.5	30.53	(13.86, 42.92)	30.37	(11.98, 42.46)	0.16	(−0.03, 0.33)
Okayama	92nd	29.0	26.65	(7.01, 40.51)	26.48	(8.08, 39.86)	0.17	(−0.17, 0.45)
Hiroshima	92nd	28.6	22.41	(1.40, 37.03)	22.15	(0.45, 37.94)	0.26	(−0.19, 0.63)
Yamaguchi	90th	27.3	32.26	(17.73, 43.58)	32.04	(17.41, 43.59)	0.22	(−0.15, 0.59)
Tokushima	94th	28.6	21.33	(−2.47, 36.31)	21.26	(−1.15, 37.81)	0.07	(−0.09, 0.23)
Kagawa	92nd	29.0	25.02	(1.10, 39.39)	24.91	(2.84, 40.62)	0.11	(−0.12, 0.34)
Ehime	91st	28.2	24.22	(6.47, 37.36)	24.10	(4.72, 37.45)	0.12	(−0.22, 0.41)
Kochi	84th	26.6	25.73	(4.70, 40.07)	25.61	(6.53, 39.40)	0.12	(−0.55, 0.76)
Fukuoka	97th	30.3	29.48	(9.48, 44.58)	29.47	(9.60, 43.84)	0.00	(−0.13, 0.13)
Saga	93rd	28.9	25.26	(4.31, 40.28)	25.18	(2.27, 40.55)	0.08	(−0.15, 0.29)
Nagasaki	90th	27.9	26.03	(5.99, 40.30)	26.00	(6.73, 40.17)	0.03	(−0.24, 0.26)
Kumamoto	92nd	28.8	18.99	(−3.51, 33.97)	18.87	(−3.43, 35.43)	0.12	(−0.20, 0.39)
Oita	91st	27.8	20.11	(−1.72, 34.72)	19.93	(12.19, 35.61)	0.18	(−0.12, 0.45)
Miyazaki	90th	27.7	16.02	(−6.27, 32.29)	15.94	(−8.13, 31.65)	0.08	(−0.31, 0.43)
Kagoshima	99th	30.5	27.38	(1.64, 44.74)	27.40	(1.62, 44.74)	−0.02	(−0.07, 0.03)
Okinawa	76th	28.1	27.41	(−9.22, 47.26)	24.48	(−13.04, 43.01)	2.93	(−0.61, 5.30)
Total	92nd		23.93	(20.15, 26.19)	23.64	(19.76, 25.87)	0.29	(0.21, 0.35)

The prefecture-specific minimum morbidity percentile (MMP) and temperature (MMT) and fraction (%) of OHCA attributable to temperature in each prefecture. The attributable fraction is expressed as the total (and as separate components) attributable to low and high temperatures with a 95% empirical confidence interval.

**Table 3:** The pooled attributable OHCA fraction computed as total and as separate components for low and high temperatures, stratified by gender and age.

Characteristics	Total (%)		Low temperature (%)		High temperature (%)		
Gender							
Male	21.12	(16.54, 23.88)	20.87	(16.23, 23.71)	0.25	(0.13, 0.32)	
Female	26.86	(22.54, 29.08)	26.50	(22.52, 28.81)	0.36	(0.24, 0.45)	
Age (years)							
18‒64	17.93	(8.82, 20.89)	15.96	(7.34, 19.15)	1.97	(−1.52, 3.59)	
65‒74	25.24	(16.39, 28.88)	24.84	(16.54, 28.53)	0.40	(−1.08, 0.85)	
75‒110	28.39	(25.87, 30.12)	28.10	(25.64, 29.77)	0.30	(0.09, 0.47)	

The attributable fraction is expressed as the total (and as separate components) attributable to low and high temperatures with a 95% empirical confidence interval.
