# Supplementary material for: Out-of-hospital cardiac arrest risk attributable to temperature in Japan
Source: Sci Rep. 2017 Jan 3;7:39538. doi: 10.1038/srep39538 (PMC5206734; doi:10.1038/srep39538)
Supplement: Supplementary Information [file srep39538-s1.pdf]

Supplementary information

Out-of-hospital cardiac arrest risk attributable to temperature in Japan

Daisuke Onozuka, PhD,[1] and Akihito Hagihara, DMSc, MPH[1]

[1] Department of Health Communication, Kyushu University Graduate School of Medical Sciences, Fukuoka, Japan

Supplementary Figure S1. Overall cumulative exposure-response associations between the relative risks (with 95% eCI, shaded grey) of OHCA and temperatures in the 47 Japanese prefectures. Exposure-response associations are expressed as the best linear unbiased prediction with the related temperature distributions. Vertical lines represent the percentile of minimum morbidity temperature (dotted) and the 2.5th and 97.5th percentiles of the temperature distribution (dashed).

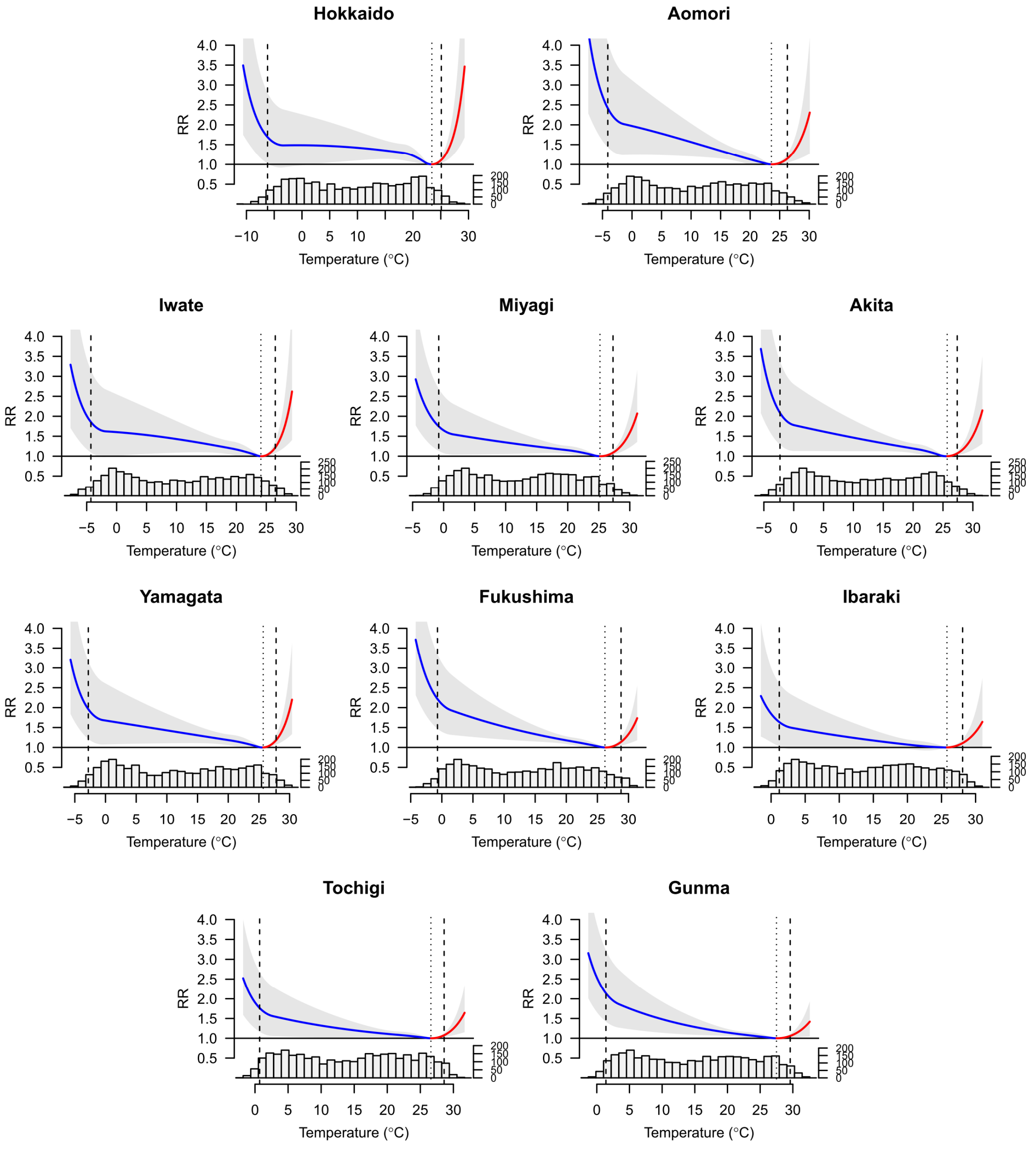

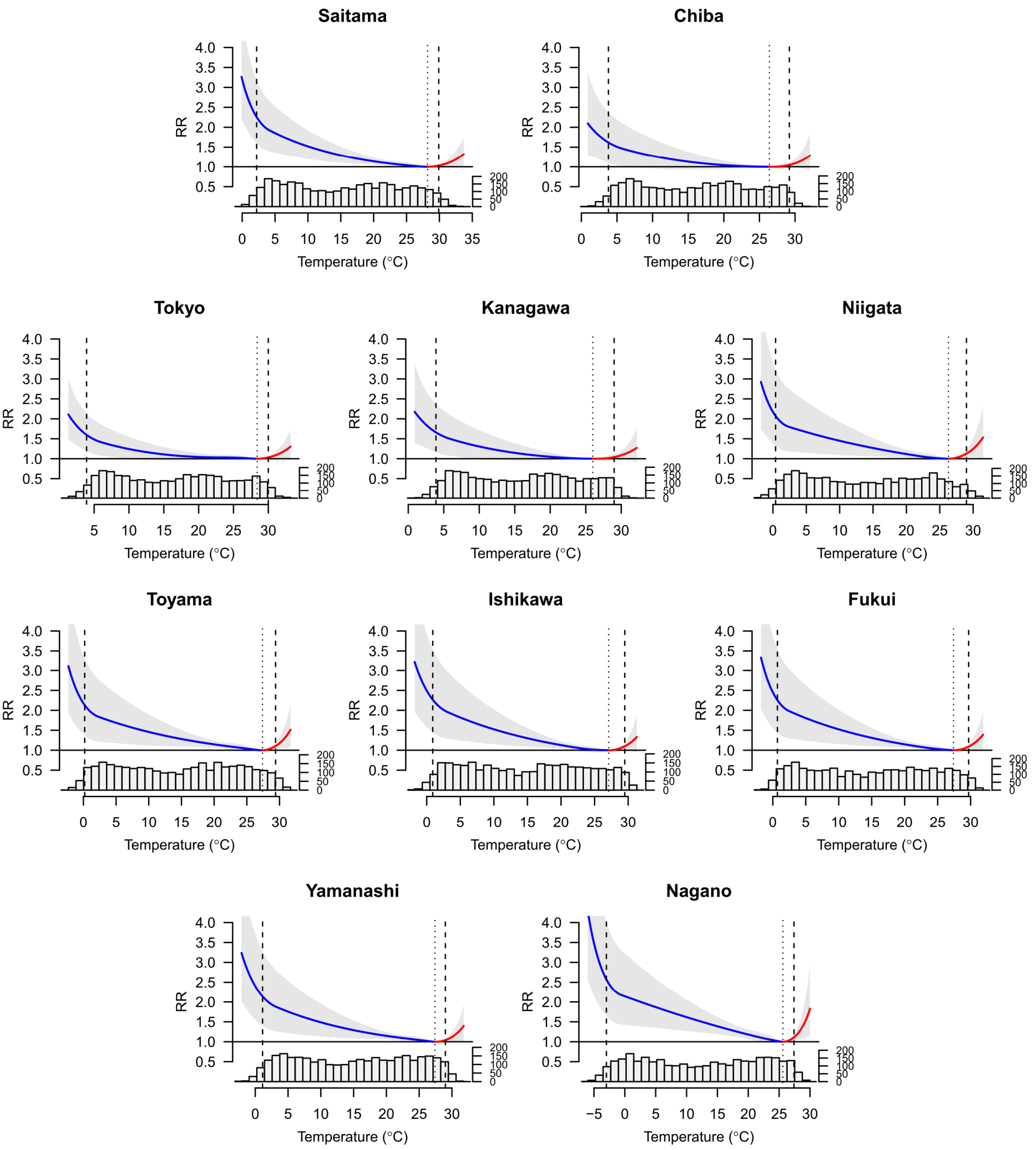
Saitama
Chiba
Tokyo
Kanagawa
Niigata
Toyama
Ishikawa
Fukui
Yamanashi
Nagano
RR
Temperature (°C)

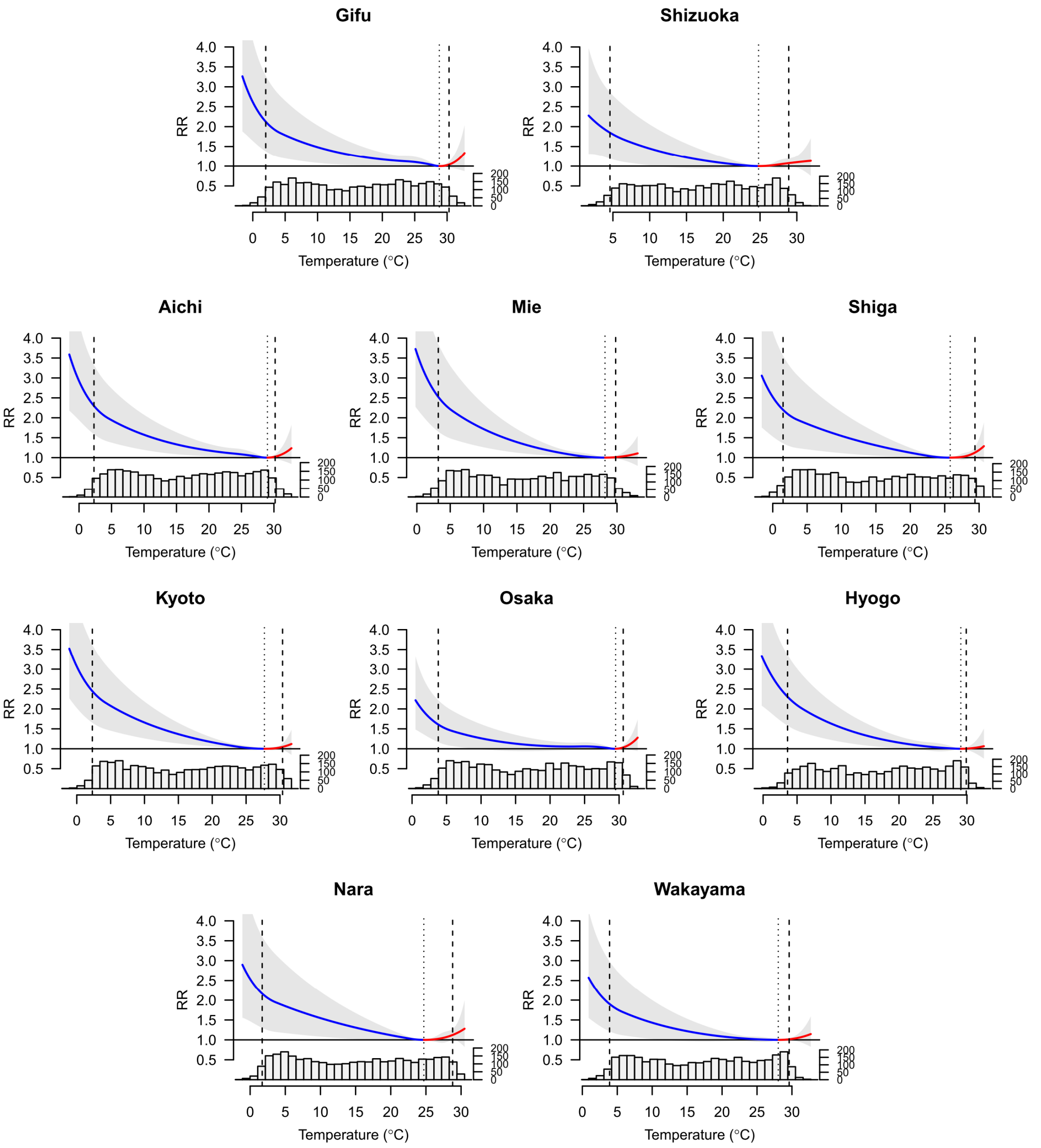

Gifu
Shizuoka
Aichi
Mie
Shiga
Kyoto
Osaka
Hyogo
Nara
Wakayama
RR
4.0
3.5
3.0
2.5
2.0
1.5
1.0
0.5
200
150
100
50
0
0
5
10
15
20
25
30
Temperature (°C)

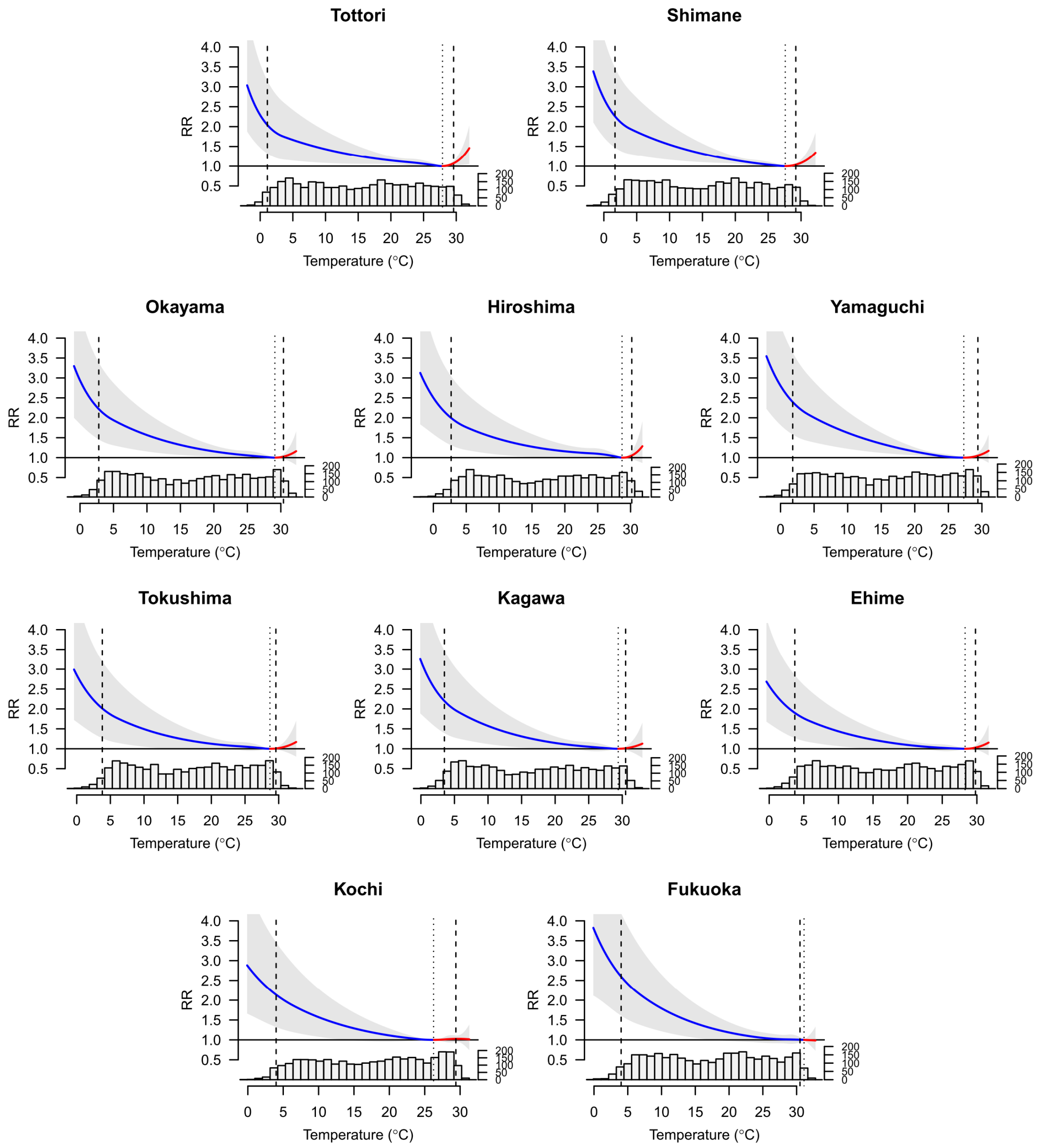

Tottori
Shimane
Okayama
Hiroshima
Yamaguchi
Tokushima
Kagawa
Ehime
Kochi
Fukuoka
RR
Temperature (°C)

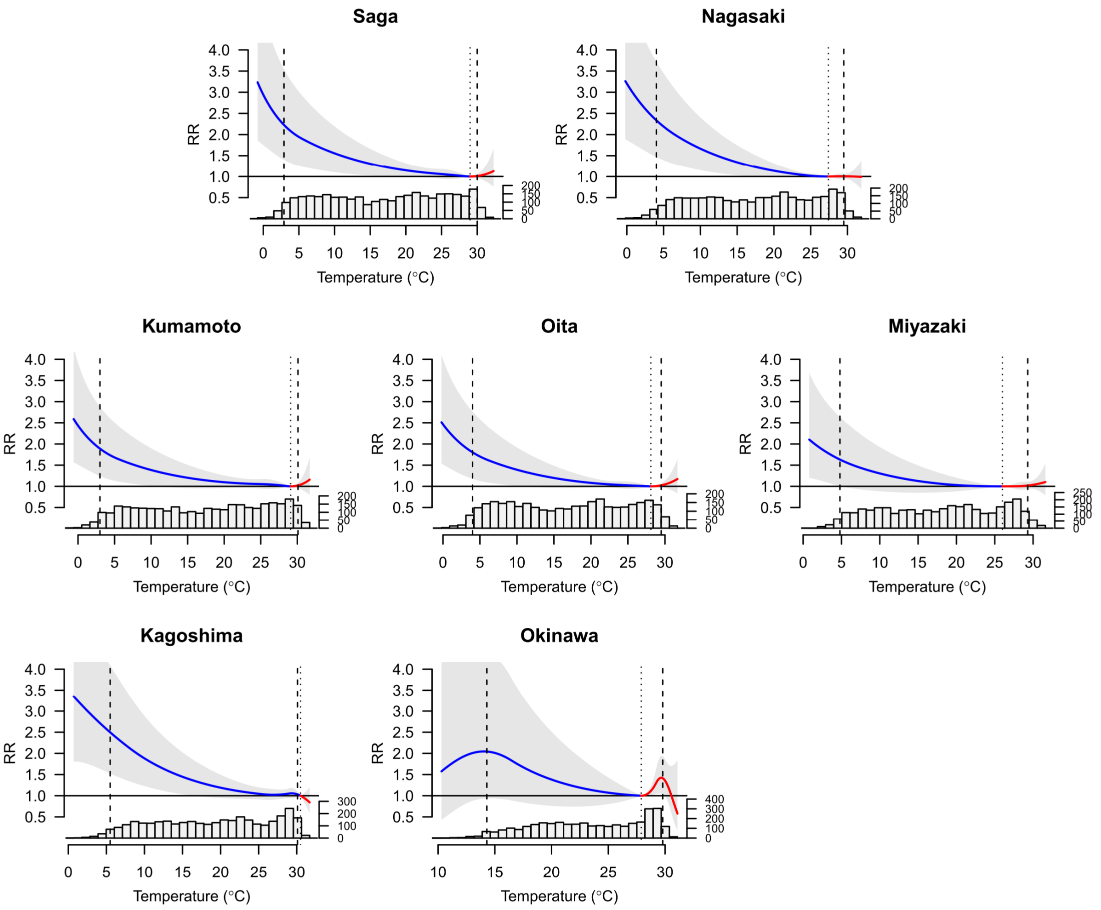
Saga
Nagasaki
Kumamoto
Oita
Miyazaki
Kagoshima
Okinawa
RR
Temperature (°C)

Supplementary Table S1. Attributable fraction (with 95% eCI) for OHCA according to prefecture expressed as the total (and as separate components) for moderate, extremely low, and high temperatures. The prefecture-specific fraction of OHCA attributable to temperature in each prefecture is expressed as separate components attributable to extreme or moderate low and high temperatures.

Prefecture	Extremely low temperature (%)		Moderately low temperature (%)		Moderately high temperature (%)		Extremely high temperature (%)	
Hokkaido	1.67	(0.36, 2.46)	25.18	(1.07, 40.88)	0.08	(0.00, 0.15)	0.45	(0.17, 0.65)
Aomori	2.24	(1.42, 2.75)	33.38	(16.32, 45.84)	0.16	(0.00, 0.33)	0.54	(0.19, 0.84)
Iwate	1.91	(0.78, 2.58)	27.85	(6.74, 43.68)	0.24	(0.06, 0.43)	0.61	(0.24, 0.90)
Miyagi	1.58	(0.48, 2.28)	20.27	(-0.02, 34.96)	0.10	(0.02, 0.17)	0.38	(0.18, 0.56)
Akita	1.87	(0.92, 2.46)	26.46	(7.06, 41.13)	0.07	(-0.02, 0.15)	0.32	(0.12, 0.49)
Yamagata	1.99	(0.91, 2.63)	28.57	(8.33, 41.87)	0.20	(0.04, 0.36)	0.53	(0.18, 0.80)
Fukushima	2.30	(1.41, 2.85)	30.43	(14.67, 41.55)	0.13	(0.01, 0.24)	0.35	(0.09, 0.55)
Ibaraki	2.10	(0.65, 2.90)	22.90	(2.77, 36.57)	0.21	(-0.14, 0.50)	0.34	(0.03, 0.60)
Tochigi	1.86	(0.71, 2.60)	19.18	(1.40, 34.01)	0.12	(0.02, 0.21)	0.28	(0.11, 0.43)
Gunma	2.15	(1.21, 2.68)	25.59	(9.27, 37.51)	0.08	(-0.03, 0.18)	0.25	(0.02, 0.44)
Saitama	2.01	(1.34, 2.47)	21.94	(6.68, 33.40)	0.06	(-0.04, 0.15)	0.18	(0.02, 0.33)
Chiba	1.67	(0.76, 2.36)	17.71	(2.16, 30.37)	0.04	(-0.04, 0.12)	0.12	(-0.06, 0.28)
Tokyo	1.48	(0.73, 1.97)	13.38	(-1.62, 24.96)	0.04	(-0.04, 0.11)	0.14	(-0.01, 0.28)
Kanagawa	1.58	(0.84, 2.12)	16.50	(1.91, 29.11)	0.07	(-0.09, 0.24)	0.13	(-0.02, 0.27)
Niigata	2.28	(1.33, 2.85)	28.28	(12.19, 40.19)	0.16	(0.01, 0.29)	0.32	(0.06, 0.54)
Toyama	2.29	(1.38, 2.90)	27.69	(10.11, 40.37)	0.14	(0.01, 0.28)	0.32	(0.05, 0.56)
Ishikawa	2.20	(1.26, 2.75)	26.68	(8.02, 39.06)	0.16	(0.00, 0.31)	0.24	(0.03, 0.43)
Fukui	2.33	(1.44, 2.95)	27.50	(9.37, 39.86)	0.16	(0.00, 0.29)	0.28	(0.03, 0.51)
Yamanashi	2.30	(1.34, 2.96)	23.72	(4.30, 36.20)	0.07	(-0.01, 0.16)	0.19	(0.04, 0.34)
Nagano	2.64	(1.86, 3.14)	34.56	(19.93, 45.34)	0.17	(0.01, 0.31)	0.38	(0.12, 0.62)
Gifu	1.99	(0.85, 2.73)	19.78	(-2.12, 34.77)	0.05	(-0.06, 0.16)	0.20	(-0.08, 0.43)
Shizuoka	1.74	(0.55, 2.44)	19.07	(-0.44, 34.06)	0.25	(-0.55, 1.01)	0.14	(-0.14, 0.40)
Aichi	2.15	(1.40, 2.64)	25.40	(8.13, 37.88)	0.02	(-0.05, 0.10)	0.14	(-0.13, 0.36)
Mie	2.11	(1.36, 2.62)	26.48	(9.43, 40.01)	0.02	(-0.06, 0.11)	0.11	(-0.10, 0.29)

Shiga	2.28	(1.33,	2.86)	26.11	(6.57,	39.46)	0.14	(-0.05,	0.33)	0.24	(0.05,	0.39)
Kyoto	2.24	(1.54,	2.67)	28.06	(14.02,	39.33)	0.08	(-0.05,	0.20)	0.16	(-0.10,	0.37)
Osaka	1.41	(0.59,	1.99)	12.01	(-5.41,	25.90)	0.04	(-0.04,	0.13)	0.16	(-0.04,	0.35)
Hyogo	2.49	(1.76,	2.99)	30.64	(12.89,	43.77)	0.00	(0.00,	0.00)	0.00	(-0.02,	0.02)
Nara	2.15	(1.08,	2.72)	29.28	(10.35,	42.45)	0.50	(-0.44,	1.29)	0.35	(-0.14,	0.73)
Wakayama	1.79	(0.86,	2.45)	19.95	(0.52,	34.31)	0.03	(-0.12,	0.18)	0.08	(-0.08,	0.22)
Tottori	1.98	(0.86,	2.64)	21.14	(0.43,	36.05)	0.12	(0.02,	0.23)	0.25	(0.05,	0.41)
Shimane	2.24	(1.40,	2.76)	28.39	(10.93,	41.10)	0.06	(-0.04,	0.15)	0.11	(-0.02,	0.22)
Okayama	2.27	(1.29,	2.88)	24.47	(6.43,	36.90)	0.05	(-0.05,	0.15)	0.13	(-0.12,	0.35)
Hiroshima	1.84	(0.79,	2.55)	20.45	(-0.52,	35.96)	0.06	(-0.07,	0.19)	0.21	(-0.12,	0.48)
Yamaguchi	2.40	(1.65,	2.88)	29.84	(14.91,	41.26)	0.09	(-0.09,	0.26)	0.14	(-0.11,	0.36)
Tokushima	1.94	(0.79,	2.65)	19.50	(-2.60,	35.06)	0.01	(-0.03,	0.05)	0.06	(-0.07,	0.18)
Kagawa	2.23	(1.09,	2.94)	22.85	(0.72,	36.83)	0.03	(-0.05,	0.10)	0.08	(-0.09,	0.25)
Ehime	2.08	(1.05,	2.73)	22.32	(3.40,	35.46)	0.03	(-0.09,	0.14)	0.09	(-0.15,	0.31)
Kochi	2.61	(1.29,	3.36)	23.39	(4.59,	37.84)	0.07	(-0.46,	0.55)	0.04	(-0.21,	0.26)
Fukuoka	2.26	(1.41,	2.74)	27.37	(8.15,	40.97)	0.00	(-0.00,	0.00)	0.00	(-0.13,	0.13)
Saga	2.54	(1.31,	3.26)	22.96	(2.84,	37.22)	0.02	(-0.06,	0.09)	0.06	(-0.12,	0.21)
Nagasaki	2.07	(1.20,	2.59)	23.93	(5.24,	37.03)	0.01	(-0.12,	0.14)	0.02	(-0.13,	0.15)
Kumamoto	2.16	(1.05,	2.97)	16.96	(-4.19,	31.29)	0.03	(-0.07,	0.13)	0.10	(-0.13,	0.30)
Oita	1.76	(0.64,	2.43)	18.27	(-2.33,	32.64)	0.05	(-0.06,	0.16)	0.13	(-0.09,	0.33)
Miyazaki	1.56	(0.42,	2.29)	14.51	(-8.71,	29.59)	0.02	(-0.13,	0.17)	0.06	(-0.21,	0.33)
Kagoshima	2.40	(1.45,	3.00)	25.29	(1.91,	42.77)	0.00	(0.00,	0.00)	0.00	(-0.01,	0.00)
Okinawa	1.80	(0.09,	2.55)	22.78	(-9.58,	42.32)	2.58	(-0.42,	4.91)	0.50	(-0.53,	1.19)
Total	1.92	(1.73,	2.01)	21.86	(18.10,	24.21)	0.10	(0.06,	0.14)	0.20	(0.15,	0.23)

Supplementary Table S2. Sensitivity analysis using the fraction (with 95% eCI) attributable to temperature (total, high and low temperature components) by varying the modelling choices and controlling for different degrees of freedom (df) for the seasonal and long-term trends (6 and 10 df per year) and relative humidity.

	Total (%)			Low temperature (%)			High temperature (%)		
Modelling choices (47 prefectrures)									
Main model	23.93	(20.15,	26.19)	23.64	(19.76,	25.87)	0.29	(0.21,	0.35)
Df/year for seasonal control: 6	25.86	(22.89,	27.81)	25.38	(22.47,	27.26)	0.48	(0.33,	0.61)
Df/year for seasonal control: 10	22.96	(18.95,	25.11)	22.50	(18.71,	24.89)	0.46	(0.32,	0.58)
Control for relative himidity (47 prefectrures)									
Main model	23.93	(20.15,	26.19)	23.64	(19.76,	25.87)	0.29	(0.21,	0.35)
With relative humidity	24.24	(20.35,	26.52)	23.97	(19.99,	26.36)	0.28	(0.20,	0.33)
